# Pyloric balloon occlusion-assisted endoscopic ultrasound-guided gastroenterostomy for an impassable duodenal stricture

**DOI:** 10.1055/a-2852-7247

**Published:** 2026-05-05

**Authors:** Dan Wang, Jiayi Ma, Ting Yang, Kaixuan Wang

**Affiliations:** 1 Digestive Endoscopy Center, Shanghai Changhai Hospital 12521 Naval Medical UniversityShanghaiChina; 2 Department of Gastroenterology, Shanghai Changhai Hospital 12521 Naval Medical UniversityShanghaiChina

**Fig. 1:**
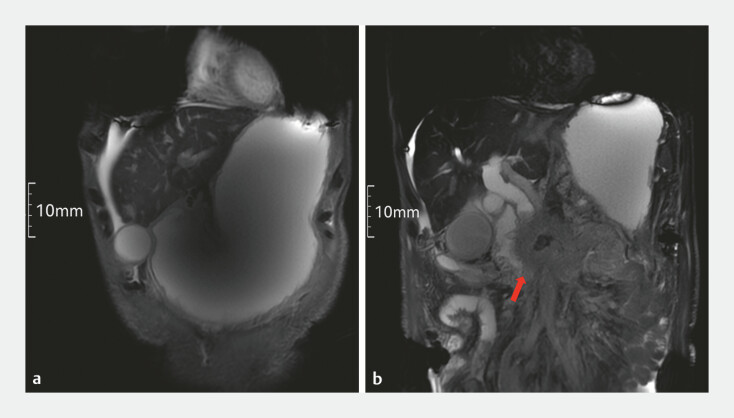
**a** Contrast-enhanced magnetic resonance imaging demonstrated gastric retention. **b** Contrast-enhanced magnetic resonance imaging showed a pancreatic head/body tumor invading the horizontal duodenum.

A 49-year-old male with a 3-month history of pancreatic cancer presented with 1 month of nausea and vomiting. Contrast-enhanced magnetic resonance imaging revealed gastric retention ( Fig. 1 **a** ) and a pancreatic head/body tumor invading the horizontal duodenum, superior mesenteric artery and vein ( Fig. 1 **b** ). Endoscopic ultrasound guided gastroenterostomy (EUS-GE) was planned to address gastric outlet obstruction.

**Fig. 2:**
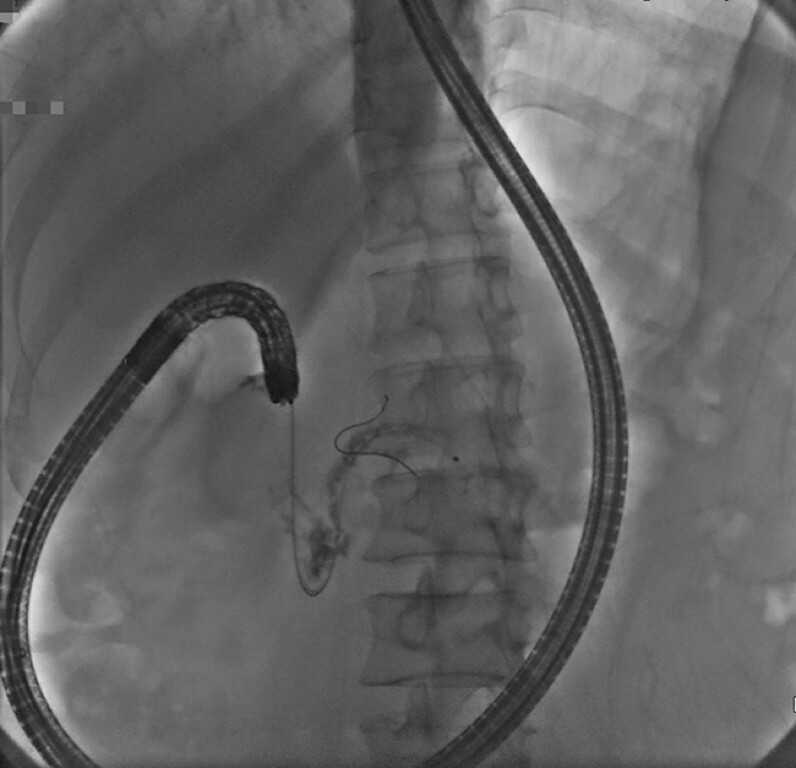
The guidewire failed to cross the stricture of the horizontal duodenum.

Intraoperatively, a contrast catheter (Boston Scientific, Marlborough, Massachusetts, USA) was advanced over a guidewire (Boston Scientific, Marlborough, Massachusetts, USA) into the descending duodenum. Enterography confirmed severe horizontal duodenal stenosis, with the guidewire unable to cross the stricture ( Fig. 2 ). The endoscope was withdrawn while the guidewire was retained.

**Fig. 3:**
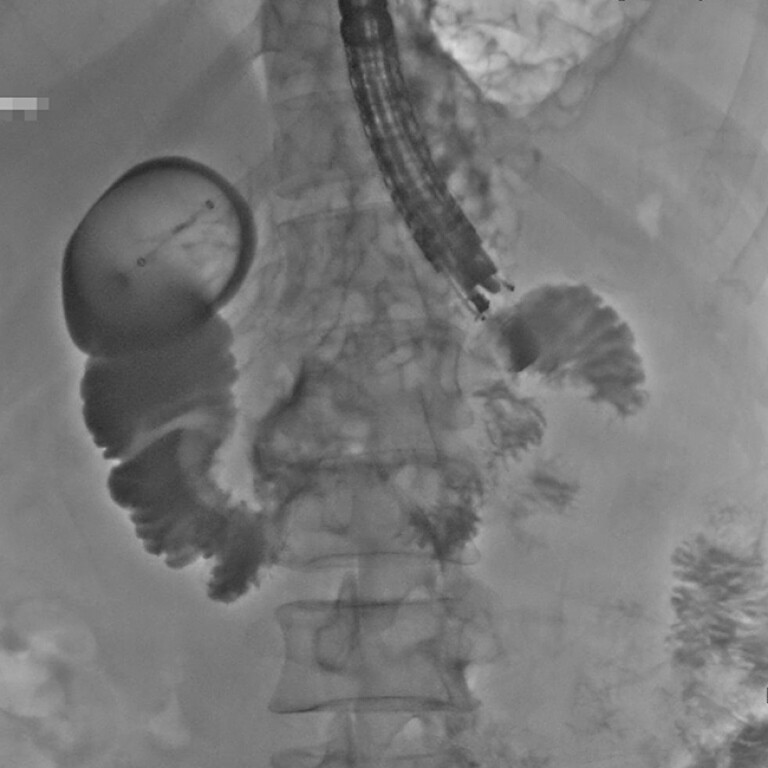
The distal duodenum and proximal jejunum were gradually filled after injection of contrast medium through the balloon’s proximal port.

**Fig. 4:**
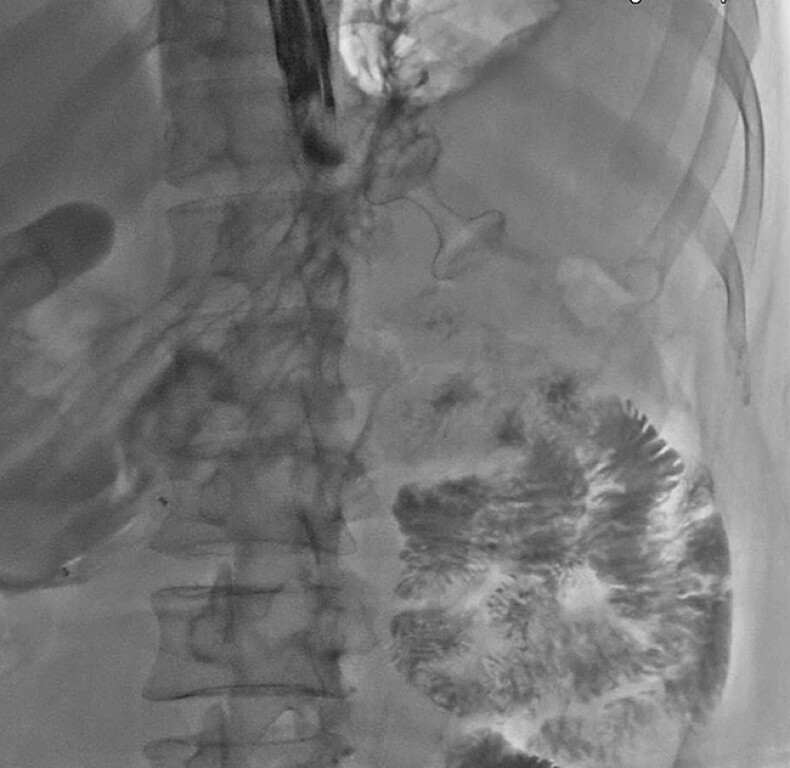
A lumen-apposing metal stent was successfully implanted.

A novel modified technique was then applied. Under the guidance of a colonoscope with a transparent cap, an occlusion balloon (Micro-Tech, Nanjing, China; maximum diameter 4.6 cm) was placed along the guidewire in the duodenum. The colonoscope was subsequently withdrawn, and a curved linear array echo endoscope was used for subsequent EUS-guided steps. The balloon was retracted to occlude the pylorus and inflated to 4.2 cm in diameter with 40 mL of air. About 300 mL of methylene blue-saline contrast was injected via the balloon’s proximal port, gradually filling the distal duodenum and proximal jejunum ( Fig. 3 ). Under EUS guidance, a dilated intestinal loop adjacent to the lower gastric body was clearly identified. A lumen-apposing metal stent was successfully implanted using an electrocautery-enhanced delivery system (Hot AXIOS System, ∅15 mm; Boston Scientific, Marlborough, Massachusetts, USA; Fig. 4 ).

Malignant duodenal stenosis with impassable strictures represents a major challenge in EUS-GE, as conventional techniques require distal balloon positioning 1 . This pyloric balloon occlusion technique, which distends the jejunum via hydrostatic pressure, is a simple, safe and effective modification for failed standard guidewire-based EUS-GE ( Video 1 ). Notably, this approach did not fully fill the small bowel lumen, making the procedure more technically challenging than standard EUS-GE.

Pyloric balloon occlusion-assisted endoscopic ultrasound-guided gastroenterostomy for an impassable duodenal stricture.Video 1

Endoscopy_UCTN_Code_TTT_1AS_2AK
